# Influential factors on quality of life in married Iranian women during the COVID-19 pandemic in 2020: a path analysis

**DOI:** 10.1186/s12905-020-01114-2

**Published:** 2021-03-10

**Authors:** Zahra Daneshfar, Shahideh Jahanian Sadatmahalleh, Samaneh Youseflu, Mahnaz Bahri Khomami, Anoshiravan Kazemnejad

**Affiliations:** 1 Department of Reproductive Health and Midwifery, Faculty of Medical Sciences, Tarbiat Modares University, Tehran, Iran; 2 Department of Midwifery, School of Nursing and Midwifery, Zanjan University of Medical Science, Zanjan, Iran; 3 Monash Centre for Health Research and Implementation, School of Public Health and Preventive Medicine, Monash University, Clayton, VIC Australia; 4 Department of Biostatistics, Faculty of Medical Sciences, Tarbiat Modares University, Tehran, Iran

**Keywords:** Quality of life, Married women, COVID-19, Path analysis

## Abstract

**Background:**

This study aimed to investigate the relationship between quality of life (QoL) with anxiety, depression, corona disease anxiety, sexual function (SF), and marital satisfaction (MS) in married women during the Covid-19 pandemic.

**Methods:**

We performed a cross-sectional study involving n = 296 married women. We used the Short Form Health Survey (SF-12), Marital Satisfaction Scale (MSS), Female Sexual Function Index (FSFI), Hospital Anxiety and Depression Scale, and coronary disease anxiety questionnaire, as determinants of QoL for data collection. Data were analyzed using the Pearson correlation coefficient and path analysis.

**Results:**

There was a relationship between the components of QoL with SF, anxiety, depression, MS, general health, and contamination obsessions. The results of path analysis also showed that that SF, MS, anxiety, general health, and corona-related anxiety have a direct effect on women’s QoL. General health has a more direct effect on QoL.

**Conclusion:**

The results of this study could help in a plan to improve the QoL of women during the coronavirus epidemic.

**Supplementary information:**

**Supplementary information** accompanies this paper at 10.1186/s12905-020-01114-2.

## Background

Coronaviruses are enveloped RNA viruses that are transmissible among humans, other mammals, and birds [1]. A novel contagious primary atypical (viral) pneumonia was reported in Wuhan, China, in December 2019. Then it was classified as a zoonotic coronavirus, similar to the SARS coronavirus and MERS coronavirus, and was named COVID-19 [2]. The World Health Organization (WHO) has recently announced COVID-19 has become a pandemic, with more than 180,000 reported cases to date [3]. The pandemic announcement raised major concerns universally with transmission prevention being comprehensively upgraded.

The uncertainty and low predictability of COVID-19 not only threaten people’s physical health but also affect people’s mental health, especially in the field of emotions and cognition. According to the Behavioral Immune System (BIS) theory, people are likely to develop negative emotions (e.g., aversion, anxiety, etc.), and negative cognitive assessment to protect themselves when faced with potential illness. Long-term negative emotions may reduce the immune function of people and disturb the balance of their normal physiological mechanisms [4]. As an outcome of the increasing number of confirmed cases and deaths caused by the COVID-19 virus, both the medical staff and members of the community have experienced psychological problems such as anxiety, depression, and stress [5].

Anxiety about COVID-19 infection is common. Coronavirus has also spread in Iran and quickly endangered mental and physical health [6]. Depression is a common mental health condition that affects many aspects of daily life and is associated with many mental health conditions, especially anxiety [7].Existing studies show that dysfunction in daily routine life and psychological problems can negatively affect the quality of life (QoL) [8]. QoL is defined by WHO as “the perception of the individual about his position in life, in the context of culture and value systems in which he lives, and about his goals, expectations, standards, and concerns [9]. Also, Sexual Function (SF) is an important element of QoL [10]. Diseases can lead to reduced QoL in various psychological and physical dimensions [11].

Considering the prevalence of the COVID-19, its various effects (physical, psychological, social, and economic effects) on people's lives, and the little information about this field, it is necessary to identify influential factors on QoL in married Iranian women during the pandemic of COVID-19.This study aimed to test a conceptual model considering the interrelated role of anxiety, depression, marital satisfaction (MS), mental health, SF, and corona disease anxiety on the QoL of married women. Based on the above aims, this study proposes the following hypotheses (Hypotheses 1–5):

### Hypothesis 1

A higher level of anxiety and depression will be associated with a lower level of QoL, SF, general health, and MS.

### Hypothesis 2

A lower level of general health will be associated with a higher level of anxiety, depression, and lower level of SF, MS, and QoL.

### Hypothesis 3

Contamination obsession will be associated with a higher level of anxiety, depression, and also have a worse effect on SF, MS, general health, and QoL.

### Hypothesis 4

The longer quarantine will be associated with a higher level of anxiety, depression, and also have a worse effect on SF, MS, and QoL.

### Hypothesis 5

SF, MS, anxiety, depression, general health, duration of quarantine, contamination obsession will be associated with QoL.

## Methods

### Design and data collection

Of the 325 women who completed the questionnaires, 29 women were excluded from the study due to a lack of inclusion criteria or incomplete filling of questionnaires. Finally, the current cross-sectional study was conducted on 296 married women.

Data collection was performed using an online questionnaire. Due to home quarantine to prevent coronavirus disease, first, the existing valid questionnaires link was designed and the designed link via social media with the help of the research team was sent to all social groups that were only women. On the first page of the link, information about the study objectives, methods, and potential outcomes was provided.

The sampling method was convenience sampling. Inclusion criteria were age range of 18–45 years, absence of the history of chronic diseases or condition resulting in sexual dysfunction (such as cardiovascular disease, diabetes, hysterectomy, premature ovarian failure, psychiatric illnesses, infertility), not using any medications affecting the sexual response cycle (such as antihypertensive drugs, antipsychotic drugs, antidepressants, hormonal drugs) and not the addiction to narcotics and alcohol, married and living with husband, and having sexual intercourse in the past 4 weeks. 

### Measures

Socio-demographic and obstetric characteristics including women’s age, age at marriage, body mass index, province and city of residence, income amount, educational level, duration of the marriage, menstrual status, job status, gravid, para, abortion, intrauterine fetal death, history of infertility, and the number of children. For using the Persian version of the questionnaires, permission was asked.

### Quality of life

Short Form Health Survey (SF-12) includes 12 questions related to 8 dimensions (sexual performance, physical role, physical pain, general health, energy and vitality, social performance, emotional role, and mental health) which are divided into two subscales of physical and mental health. The maximum score obtained for each section or subscale is 100 and the minimum score is zero, that a higher score indicating a better health status. The validity and reliability of this questionnaire were previously confirmed in Iran (Additional file 1) [12].

### Sexual function

The Female Sexual Function Index (FSFI) is a multidimensional self-report tool for evaluating the main dimensions of SF in women (sexual desire, arousal, lubrication, orgasm, satisfaction, and pain) with 19 items [13]. Its validity and reliability in Iran were previously confirmed (Additional file 1) [14].

### Depression and anxiety

Hospital Anxiety and Depression Scale (HADS) designed by Zigmond and his colleagues in 1983, has seven questions on symptoms of anxiety and seven questions on symptoms of depression. This questionnaire is based on a four-point Likert scale. Finally, out of a total of 21-points score, scores above 8 were considered as being anxious and depressed in each subscale. This scale has been validated as a good tool for screening mental health disorders in Iran (Additional file 1) [15]**.**

### Marital satisfaction

The Marital Satisfaction Scale-shortened version (MSS) contains 10 items measuring the satisfaction of marital relationships. Using the 5-point Likert scale, the answers range from “5 = I quite agree with” to “1 = I quite disagree with”. Questions number Q1, Q3, Q5, Q8, and Q9 were negative items and need reversing. The total scoring of this questionnaire ranges from 10 to 50. A higher score indicates a higher MS. A valid and reliable version of the MSS scale was translated into Persian by Arab Alidousti et al. The Persian version showed the desired validity and reliability (Additional file 1) [16].

### Mental health

General Health Questionnaire (GHQ) was developed by Goldberg in 1978. The GHQ-28 is a screening tool to detect those likely to have or to be at risk of developing psychiatric disorders. The GHQ-28 has been divided into four subscales. These are: somatic symptoms (items 1–7); anxiety/insomnia (items 8–14); social dysfunction (items 15–21), and severe depression (items 22–28) [17]. Psychometric of 28 item form of this questionnaire (GHQ) was conducted in Iran, that its results have confirmed validity and reliability (Additional file 1) [18].

### Corona disease anxiety

Corona disease anxiety scale has been prepared and validated by Alipour et al. to measure anxiety caused by the prevalence of the coronavirus in Iran. To prepare the Corona Anxiety Scale, questions of the AIDS anxiety questionnaire and questionnaires related to fear of health risks were surveyed and 23 items selected. The final version of this tool has 18 items and 2 components. Items 1 to 9 are for measuring psychological symptoms and items 10 to 18 are for physical symptoms. This tool is in the 4-degree Likert range (never = 0, sometimes = 1, most of the time = 2 and Always = 3). Therefore, the highest and lowest scores obtained by the respondents in this questionnaire are between 0 and 54. The high scores in this questionnaire indicate a higher level of anxiety in individuals (Additional file 1) [6].

### Statistical analysis

Software SPSS (version 20) was used for descriptive statistics and to determine the effects of variables on each other, the method of path analysis with Lisrel software was used. Correlations between the variables were examined using Pearson’s correlation coefficients.

The path analysis method is a generalized total regression that can express in addition to the direct effects, indirect effects, and the general effect of each of the variables for the dependent variables, and interpret the observed relationships and correlations between them with logical expression.

In the present study, the Root Mean Square Error of Approximation (RMSEA), goodness fit Comparative Fit Index (CFI) used to determine the fit of the model. These indicators are similar to the correlation coefficient. Their value varies between zero and one. Chi-Square value evaluates the overall model fit [19].

## Results

Table 1 describes the socio-demographic characteristics of participants. The mean age of participants and the duration of their marriage were 33.68 ± 6.47 and 10 ± 7 years, respectively. The majority of participants (63.17%) were in home quarantine for 6–7 days per week. 82.43% of the samples had academic level education and about 40.88% of them were unemployed. The overall mean score of QoL was 62 ± 7.

**Table 1:** Socio-demographic characteristics of samples

Characteristic	
Age (years)^a^	33.68 ± 6.47
Parity ^a^	1.07 (0)
Duration of marriage (years)^a^	10 (7)
Duration of quarantine^b^	
Less than 1 day/week	11 (3)
Between 2–3 day/week	28 (9)
Between 4–5 day/week	68 (22)
Between 6–7 day/week	187 (62)
At all	5 (1)
Income (Toman)^b^	
Less than 1 million	41 (11)
Between 1–3 million	103 (31)
Between 3–5 million	104 (32)
More than 5 million	74 (22)
Education^b^	
High school	56 (16)
University	268 (82.46)
Job status^b^	
Unemployed	134 (41)
Employed^b^	155 (47.69)
Self-employed	18 (5)
Student	15 (4)
QoL	62 ± 7

^a^Values are given as mean ± SD

^b^Values are given as number (%)

Table 2 shows the Correlation (bivariate analysis) between all variables included in the path model. Results showed that QoL was correlated with SF (r = 0.53, P < 0.001), anxiety (r = − 0.22, P < 0.001), depression (r = − 0.17, P < 0.01), MS (r = 0.28, p < 0.001), general health (r = − 0.52, P < 0.001), and contamination obsessions (r = − 0.14, P < 0.05).

**Table 2:** Correlations between anxiety, depression, marital satisfaction, sexual function, general health, duration of quarantine, contamination obsessions, and quality of life of women

	1	2	3	4	5	6	7
1. Quality of life	–	–	–	–	–		
2. Sexual function	0.53***	–	–	–	–		
3. Anxiety	− 0.22***	− 0.17**	–	–	–		
4. Depression	− 0.17**	− 0.07	0.26***	–	–		
5. Marital satisfaction	0.28***	0.28***	0.08	− 0.08	–		
6. General health	− 0.52***	− 0.34***	0.21***	0.28**	− 0.01		
7. Duration of quarantine	− 0.06	− 0.06	0.06	0.13*	0.03	0.11*	
8.contamination obsessions	− 0.14*	− 0.14*	0.03	0.18**	0.08	0.12*	0.25*

Values are given as Pearson coefficient (P-value) using Pearson correlation test

*P < 0.05; ** P < 0.01; *** P < 0.001

The overall goodness-of-fit statistics demonstrated that the conceptual model of the study was excellent (P-value = 0.01; chi2 = 37.82; DF = 21; chi2/df = 1.81; RMSEA = 0.05; CFI = 0.97; GFI = 0.98) (Table 3).

**Table 3:** The goodness of fit indices for the models

	CFI	GFI	RMSEA	Chi-square	df	Chi-square/df	P-value
Path N = 296	0.97	0.98	0.05	37.82	21	1.81	0.01

*CFI* comparative fit index, *GFI* goodness fit index, *RMSEA* root mean square error of approximation, *Chi-square/df* chi-square to the degree of freedom index

**Fig. 1:**
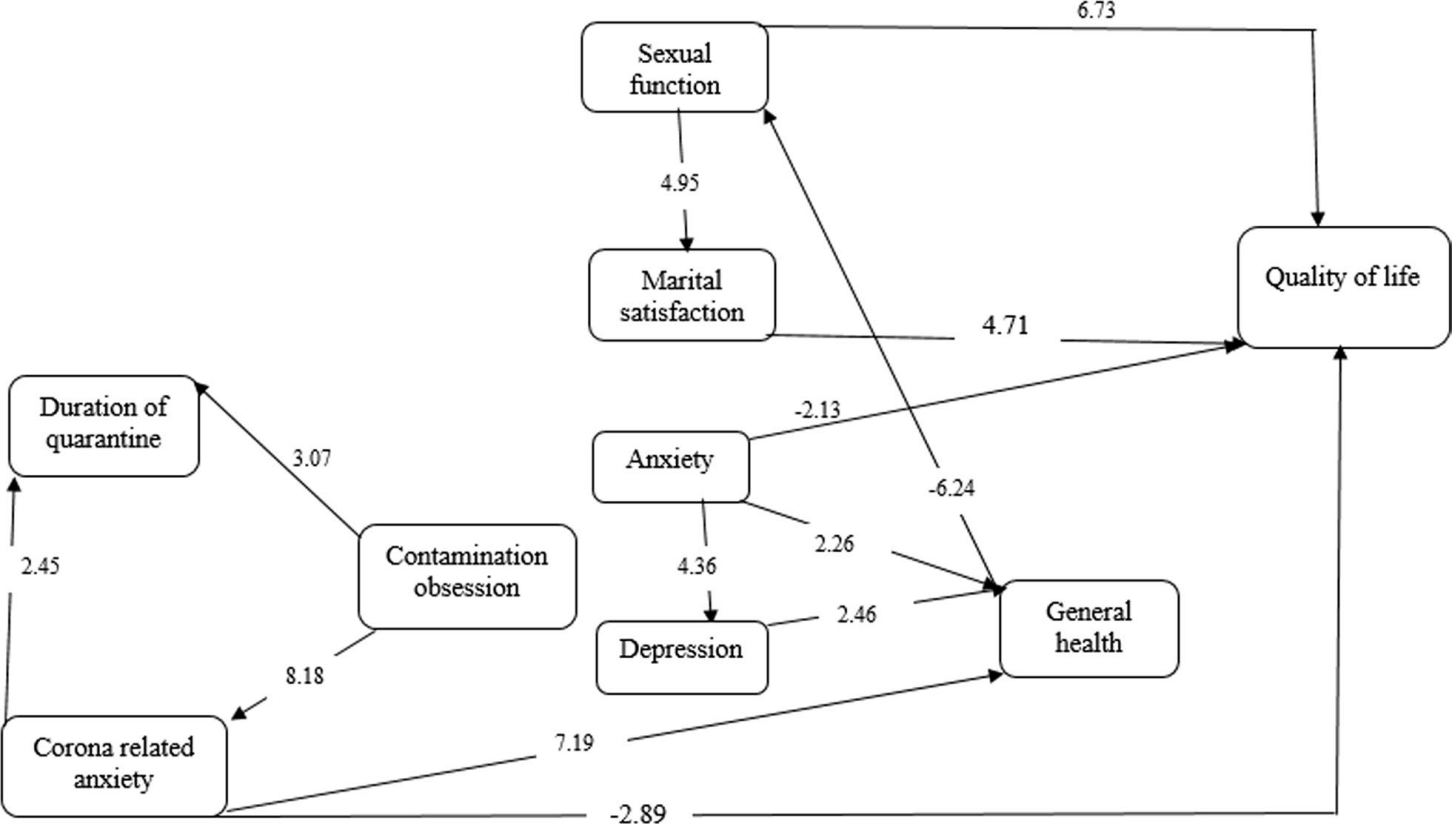
Path diagram (T-value) for the predictors of QoL

Table 4 and Fig. 1 show the direct, indirect, and total effects of variables on women's QoL. Results show that SF (β = 0.31), MS (β = 0.21), anxiety (β = − 0.09), general health (β = − 0.33), and corona-related Anxiety (β = − 0.14) have a direct effect on women’s QoL. Among variables, general health has a more direct effect on QoL. Anxiety, depression, and corona related anxiety are the main predictors of general health. On the other hand, general health with indirect effects through SF can change women's QoL. Women with a higher level of SF had better MS and QoL. We also observed a lower level of QoL and longer duration of quarantine in women with a higher level of corona related anxiety. Contamination obsessions were related to more duration of quarantine, and a higher level of corona related anxiety.

**Table 4:** Direct, indirect, and total effect of some variables on QoL of women

	Direct effect	Indirect effect	Total effect	T-value
Sexual function	0.31	0.38	0.69	7.98
Marital satisfaction	0.21	–	0.21	4.71
Anxiety	− 0.09	− 0.34	− 0.43	− 3.33
Depression	–	− 0.20	− 0.20	− 2.53
General health	− 0.33	− 0.08	− 0.41	− 8.9
Corona-related anxiety	− 0.14	− 0.15	− 0.29	− 6.55
Contamination obsessions	–	− 0.13	− 0.13	− 5.11

^*^Path values are standardized β coefficients

## Discussion

The pandemic of COVID-19 severely affects the lives of individuals around the world. Social distances have been integrated into human lives. Disruption to the social norms could therefore drive stress because of loneliness, anxiety, depression, mental disorders, health risks, and many other things that affect a person's life [20].

In this study, the effect of factors: SF, MS, mental health, anxiety, depression, corona disease anxiety on the QoL of married women during the epidemic of COVID virus 19 was studied using path analysis. Our results showed the SF, MS, anxiety, general health, and corona-related anxiety have a direct effect on women’s QoL. In some studies, sexual dysfunction has been identified as a factor influencing on QoL in individuals, and in fact, the SF plays an important role in studies related to QoL [21]. In the study of Zhang and his colleague, 52.1% of participants felt horror and apprehensive due to the pandemic of COVID-19, and the pandemic was associated with mild stressful impact in participants [22]. SF may be affected by any type of stress or emotional distress [23]. Therefore, it can be said that stress caused by COVID-19 can affect SF and SF is also one of the factors affecting the QoL.

In our study, MS was identified as an influential factor on QoL and the results of a study by Keramat et al. confirmed this finding. In the study of Keramat, a significant relationship was found between QoL and MS. The study was performed on infertile women [24]. Infertility and its treatments, like Corona's pandemic, is a crisis that can affect the MS and then the QoL changes. In studies, MS and SF have been associated with QoL [25]. One of the indicators of mental health is MS [26], so it can affect the QoL.

Anxiety and depression are the most important factors influencing the QoL associated with health and even its impact on QoL have been reported to be stronger than physical conditions such as angina and other chronic diseases [27]. Anxiety about COVID-19 is common and seems to be mostly due to uncertainties. Low scientific knowledge about the COVID-19 also exacerbates this anxiety [6]. In this study, corona disease anxiety and anxiety had a direct impact on QoL that was in line with the results of a study by Parsamehr et al. In this study, there was a significant negative relationship between depression and anxiety with patients' QoL who had been undergone coronary artery bypass graft. The researchers said anxiety and depression can reduce the QoL of patients by causing physical and psychological consequences [28]. Anxiety is a natural reaction to difficult and threatening situations, but if it is severe and prolonged and causes suffering, it disrupts life and reduces the QoL.

In addition to causing physical harm, COVID-19 also has a serious effect on people's mental health [29]. In our study, among the variables affecting QoL, general health has a strong direct impact. QoL has a multidimensional and complex meaning and often as a specific understanding of satisfaction in life, physical health, social and family health, hope, social etiquette, and mental health [30]. It is believed that various factors might influence the QoL that mental health is one of the important and influential factors in QoL [31].

Anxiety and depression are two important aspects of mental health [32]. In the present study, anxiety, depression, and corona related anxiety are the main predictors of general health. The results of a study conducted by Alizadeh Fard et al. confirmed our findings. In Alizadeh's study, corona disease anxiety was negatively correlated with mental health. Anxiety is the most fundamental feature of a crisis. The critical condition of the COVID-19 pandemic by increasing negative factors such as anxiety affects mental health [33]. Women are the most vulnerable group in society to mental health problems during the outbreak of Covid-19. One of the main concerns of women in this period is receiving reproductive-sexual, pregnancy, childbirth, and postpartum care which has involved a large number of them. Women in pregnancy and during postpartum may represent a vulnerable population who might be severely psychologically affected [34]. Social distancing and quarantine restrictions can increase anxiety in most pregnant mothers during the spread of COVID-19 and on the other hand, they are worried about their children after birth for vaccinations and necessary screenings in pandemic conditions. Therefore, the protocols and guidelines for the management of COVID- 19 in the field of obstetrics and gynecology should be improved.

Epidemics of the diseases affect people physically as well as society at different levels which leads to mental disorders such as anxiety and stress. In our study, depression was also a predictor of general health. In a study conducted by Steger et al., depression is considered to be the strongest predictor of mental health [35]. The results of the study by Wu et al. showed that awareness of COVID-19 significantly increased the prevalence of perinatal depression [36]. Also, in the study of Zheng et al., they concluded that the prevalence of COVID-19 may be higher than the incidence of depression in cancer patients [37]. It seems that excessive stress and anxiety in the current situation increase the risk of depressive disorder.

In our study, women with a higher level of SF had better MS. This result is congruent with the study of Khazei et al. In this study, there was a significant relationship between sexual dysfunction and low MS. MS is related to the level and quality of general health and life satisfaction [38]. MS is impressed by many factors. A Safe and enjoyable sexual relationship is one of the most important factors considered in many studies [39]. Sexual dysfunction probably affects MS through its impact on QoL and mental health.

In this study, more duration of quarantine will be associated with a higher level of anxiety, depression, and QoL. A review of Brooks et al. showed that quarantine has negative psychological effects containing post-traumatic stress symptoms, anger, and confusion. During quarantine days, separation from loved ones, loss of liberty, uncertainty about the condition of the disease, and boredom can have impressive effects. Stressors included longer quarantine duration, infection fears, disappointment, fatigue, incomplete supplies, inadequate information, financial loss, and stigma [40].

The result of the present study showed that contamination obsessions were related to more duration of quarantine, and a higher level of corona related anxiety. Among the many psychosocial consequences that have occurred due to the pandemic of COVID-19, the prominence of obsessive–compulsive symptoms has been largely neglected. Uncontrolled obsessions and compulsions can lead to dermatological conditions, chronic stress, insomnia, and high risk for suicide [41].

### Limitations and strengths

Due to the epidemic conditions, this study was conducted through virtual networks. Some married women who did not have the ability or access to the use of virtual networks did not participate in this study. Also, because the research was done through virtual, there was no complete supervision of the research team on completing the questionnaires.

The strengths of the present study include it is the first study conducted in this field in Iran and in the world, and the participants were from a wide range of socioeconomic and geographic backgrounds.

## Conclusion

The results of this study showed that SF, MS, anxiety, general health, and corona-related anxiety are factors influencing the QoL during the pandemic of COVID-19. Stress and anxiety disturb the immune system and make individuals vulnerable to contagious diseases such as corona. This warrants research on strategies to improve people’s mental health.

## Supplementary information

**Additional file 1.** Questionnaires used in this study.

## Data Availability

The data sets used and analyzed during the current study are available from the corresponding author on reasonable request.

## Abbreviations

FSFI: Female Sexual Function Index
QOL: Quality of life
SF12: Short Form Health Survey
SARS: Severe Acute Respiratory Syndrome
MERS: Middle East Respiratory Syndrome
COVID19: Corona virus disease 2019
SF: Sexual function
MS: Marital satisfaction
WHO: World Health Organization
BIS: Behavioral immune system
BMI: Body Mass Index
HADS: Hospital Anxiety and Depression Scale
GHQ: General Health Questionnaire
MSS: Marital Satisfaction Scale-shortened version
RMSEA: Root mean square error of approximation
AGFI: Adjusted Goodness of Fit Index
CFI: Confirmatory factor analytic
DF: Degree of freedom
