# Supplementary material for: Influential factors on quality of life in married Iranian women during the COVID-19 pandemic in 2020: a path analysis
Source: BMC Womens Health. 2021 Mar 10;21:102. doi: 10.1186/s12905-020-01114-2 (PMC7944245; doi:10.1186/s12905-020-01114-2)
Supplement: Supplementary file 1 — Additional file 1. Questionnaires used in this study. [file 12905_2020_1114_MOESM1_ESM.doc]

**Short Form Health Survey (SF-12)**

1. In general, would you say your health is:

1- Excellent 2- Very good 3- Good 4 -Fair 5- Poor

The following questions are about activities you might do during a typical day. Does your health now limit you in these activities? If so, how much

Yes, a limited a lot Yes , limited a little NO, not limited at all

2. Moderate activities such as moving a table, pushing a vacuum cleaner, bowling, or playing golf.

3. Climbing several flights of stairs.

During the past 4 weeks, have you had any of the following problems with your work or other regular daily activities as a result of your physical health?

Yes NO

4. Accomplished less than you would like.

5. Were limited in the kind of work or other activities.

During the past 4 weeks, have you had any of the following problems with your work or other regular daily activities as a result of any emotional problems (such as feeling depressed or anxious)?

Yes NO

6. Accomplished less than you would like

7. Did work or activities less carefully than usual.

8. During the past 4 weeks, how much did pain interfere with your normal work (including work outside the home and housework)?

□1 Not at all □2 A little bit □3Moderately □4Quite a bit □5Extremely

These questions are about how you have been feeling during the past 4 weeks. For each question, please give the one answer that comes closest to the way you have been feeling. How much of the time during the past 4 weeks?

All of the time Most of the time A good bit of time Some of the time A little of the time None of the time

9. Have you felt calm & peaceful?

10. Did you have a lot of energy?

11. Have you felt down-hearted and blue?

12. During the past 4 weeks, how much of the time has your physical health or emotional problems interfered with your social activities (like visiting friends, relatives, etc.)?

1- All of the time 2-Most of the time 3-Some of the time 4-A little of the time 5-None of the time

**Female Sexual Function Index Scoring**

1. Over the past 4 weeks, how often did you feel sexual desire or interest?

5 = Almost always or always

4 = Most times (more than half the time)

3 = Sometimes (about half the time)

2 = A few times (less than half the time)

1 = Almost never or never

1. Over the past 4 weeks, how would you rate your level (degree) of sexual desire or interest?

5 = Very high 4 = High 3 = Moderate 2 = Low 1 = Very low or none at all

1. Over the past 4 weeks, how often did you feel sexually aroused ("turned on") during sexual activity or intercourse?

0 = No sexual activity

5 = Almost always or always

4 = Most times (more than half the time)

3 = Sometimes (about half the time)

2 = A few times (less than half the time)

1 = Almost never or neve

1. Over the past 4 weeks, how would you rate your level of sexual arousal ("turn on") during sexual activity or intercourse?

0 = No sexual activity 5 = Very high 4 = High 3 = Moderate 2 = Low 1 = Very low or none at all

1. Over the past 4 weeks, how confident were you about becoming sexually aroused during sexual activity or intercourse?

0 = No sexual activity 5 = Very high confidence 4 = High confidence 3 = Moderate confidence 2 = Low confidence 1 = Very low or no confidence

1. Over the past 4 weeks, how often have you been satisfied with your arousal (excitement) during sexual activity or intercourse?

0 = No sexual activity

5 = Almost always or always

4 = Most times (more than half the time)

3 = Sometimes (about half the time)

2 = A few times (less than half the time)

1 = Almost never or never

1. Over the past 4 weeks, how often did you become lubricated ("wet") during sexual activity or intercourse?

0 = No sexual activity

5 = Almost always or always

4 = Most times (more than half the time)

3 = Sometimes (about half the time)

2 = A few times (less than half the time)

1 = Almost never or never

1. Over the past 4 weeks, how difficult was it to become lubricated ("wet") during sexual activity or intercourse?

0 = No sexual activity

1 = extremely difficult or impossible

2 = Very difficult

3 = Difficult

4 = slightly difficult

5 = Not difficult

1. Over the past 4 weeks, how often did you maintain your lubrication ("wetness") until completion of sexual activity or intercourse?

0 = No sexual activity

5 = Almost always or always

4 = Most times (more than half the time)

3 = Sometimes (about half the time)

2 = A few times (less than half the time)

1 = Almost never or never

1. Over the past 4 weeks, how difficult was it to maintain your lubrication ("wetness") until completion of sexual activity or intercourse?

0 = No sexual activity

1 = extremely difficult or impossible

2 = Very difficult

3 = Difficult

4 = slightly difficult

5 = Not difficult

1. Over the past 4 weeks, when you had sexual stimulation or intercourse, how often did you reach orgasm (climax)?

0 = No sexual activity

5 = Almost always or always

4 = Most times (more than half the time)

3 = Sometimes (about half the time)

2 = A few times (less than half the time)

1 = Almost never or never

1. Over the past 4 weeks, when you had sexual stimulation or intercourse, how difficult was it for you to reach orgasm (climax)?

0 = No sexual activity

1 = extremely difficult or impossible

2 = Very difficult

3 = Difficult

4 = slightly difficult

5 = Not difficult

1. Over the past 4 weeks, how satisfied were you with your ability to reach orgasm (climax) during sexual activity or intercourse?

0 = No sexual activity

5 = Very satisfied

4 = moderately satisfied

3 = about equally satisfied and dissatisfied

2 = moderately dissatisfied

1 = Very dissatisfied

1. Over the past 4 weeks, how satisfied have you been with the amount of emotional closeness during sexual activity between you and your partner?

0 = No sexual activity

5 = Very satisfied

4 = moderately satisfied

3 = about equally satisfied and dissatisfied

2 = moderately dissatisfied

1 = Very dissatisfied

1. Over the past 4 weeks, how satisfied have you been with your sexual relationship with your partner?

5 = Very satisfied

4 = moderately satisfied

3 = about equally satisfied and dissatisfied

2 = moderately dissatisfied

1 = Very dissatisfied

1. Over the past 4 weeks, how satisfied have you been with your overall sexual life?

5 = Very satisfied

4 = moderately satisfied

3 = about equally satisfied and dissatisfied

2 = moderately dissatisfied

1 = Very dissatisfied

1. Over the past 4 weeks, how often did you experience discomfort or pain during vaginal penetration?

0 = did not attempt intercourse 1 = Almost always or always 2 = Most times (more than half the time) 3 = Sometimes (about half the time) 4 = A few times (less than half the time) 5 = Almost never or never

1. Over the past 4 weeks, how often did you experience discomfort or pain following vaginal penetration?

0 = Did not attempt intercourse 1 = Almost always or always 2 = Most times (more than half the time) 3 = Sometimes (about half the time) 4 = A few times (less than half the time) 5 = Almost never or never

1. Over the past 4 weeks, how would you rate your level (degree) of discomfort or pain during or following vaginal penetration?

0 = did not attempt intercourse

1 = Very high

2 = High

3 = Moderate

4 = Low

5 = Very low or none at all

**Hospital Anxiety and Depression Scale (HADS)**

**I feel tense or 'wound up':**

3-Most of the time

2-A lot of the time

1-From time to time, occasionally

0-Not at all

**I still enjoy the things I used to enjoy:**

Definitely as much

Not quite so much

Only a little

Hardly at all

**I get a sort of frightened feeling as if something awful is about to happen:**

Very definitely and quite badly

Yes, but not too badly

A little, but it doesn't worry me

Not at all

**I can laugh and see the funny side of things:**

As much as I always could

Not quite so much now

Definitely not so much now

Not at all

**Worrying thoughts go through my mind:**

A great deal of the time

A lot of the time

From time to time, but not too often

Only occasionally

**I feel cheerful**

Not at all

Not often

Sometimes

Most of the time

**I can sit at ease and feel relaxed:**

Definitely

Usually

Not at all

Not Often

**I feel as if I am slowed down:**

Nearly all the time

Very often

Sometimes

Not at all

**I get a sort of frightened feeling like 'butterflies' in the stomach:**

Not at all

Occasionally

Quite Often

Very Often

**I have lost interest in my appearance:**

Definitely

I don't take as much care as I should

I may not take quite as much care

I take just as much care as ever

**I feel restless as I have to be on the move:**

Very much indeed

Quite a lot

Not very much

Not at all

**I look forward with enjoyment to things:**

As much as I ever did

Rather less than I used to

Definitely less than I used to

Hardly at all

**I get sudden feelings of panic:**

Very often indeed

Quite often

Not very often

Not at all

**I can enjoy a good book or radio or TV program:**

Often

Sometimes

Not often

Very seldom

**Marital Satisfaction Scale-shortened version (MSS)**

Response choices

1-Strongly Disagree 2-Moderately Disagree 3-Neither Agree nor Disagree 4- Moderately Agree 5-Strongly Agree

1-I am not pleased with the personality characteristics and personal habits of my
partner.

2- I am very happy with how we handle role responsibilities in our marriage.

3- I am not happy about our communication and feel my partner does not understand me.

4- I am very happy about how we make decisions and resolve conflicts.

5- I am unhappy about our financial position and the way we make financial decisions.

6- I am very happy with how we manage our leisure activities and the time we spend together.

7- I am very pleased about how we express affection and relate sexually.

8- I am not satisfied with the way we each handle our responsibilities as parents.

9- I am dissatisfied about our relationship with my parents, in-laws, and/or friends.

10- I feel very good about how we each practice our religious beliefs and values.

**General Health Questionnaire (GHQ)**

1. Been feeling perfectly well and in good health?

2. Been feeling in need of a good tonic?

3. Been feeling run down and out of sorts?

4. Felt that you are ill?

5. Been getting any pains in your head?

6. Been getting a feeling of tightness or pressure in your head?

7. Been having hot or cold spells?

8. Lost much sleep over worry?

9. Had difficulty in staying asleep once you are off?

10. Felt constantly under strain?

11. Been getting edgy and bad-tempered?

12. Been getting scared or panicky for no good reason?

13. Found everything getting on top of you?

14. Been feeling nervous and strung-up all the time?

15. Been managing to keep yourself busy and occupied?

16. Been taking longer over the things you do?

17. Felt on the whole you were doing things well?

18. Been satisfied with the way you’ve carried out your task?

19. Felt that you are playing a useful part in things?

20. Felt capable of making decisions about things?

21. Been able to enjoy your normal day-to-day activities?

22. Been thinking of yourself as a worthless person?

23. Felt that life is entirely hopeless?

24. Felt that life isn’t worth living?

25. Thought of the possibility that you might make away with yourself?

26. Found at times you couldn’t do anything because your nerves were too bad?

27. Found yourself wishing you were dead and away from it all?

28. Found that the idea of taking your own life kept coming into your mind?

**Corona disease anxiety scale**

1-Thinking about Corona makes me anxious.

0. Never 1. Sometimes 2. Most of the time 3. Always

2. I feel tense when I think about the corona threat.

0. Never 1. Sometimes 2. Most of the time 3. Always

3. I am very worried about the prevalence of corona disease.

0. Never 1. Sometimes 2. Most of the time 3. Always

4. I'm afraid to get a corona.

0. Never 1. Sometimes 2. Most of the time 3. Always

5. I think I might get corona disease at any moment.

0. Never 1. Sometimes 2. Most of the time 3. Always

6. At the slightest sign, I think I have taken a corona and am examining myself.

0. Never 1. Sometimes 2. Most of the time 3. Always

7. I am worried about the spread of corona to those around me.

0. Never 1. Sometimes 2. Most of the time 3. Always

8. Corona anxiety disrupts daily activities.

0. Never 1. Sometimes 2. most of the time 3. Always

9. The media attention to Corona worries me.

0. Never 1. Sometimes 2. Most of the time 3. Always

10. Thinking about Corona has disturbed my sleep.

0. Never 1. Sometimes 2. Most of the time 3. Always

11. Thinking about Corona has made me lose my appetite.

0. Never 1. Sometimes 2. Most of the time 3. Always

12. I get a headache when I think of Corona.

0. Never 1. Sometimes 2. Most of the time 3. Always

13. My body trembles when I think of Corona.

0. Never 1. Sometimes 2. Most of the time 3. Always

14. When I think of Corona, my body hair stands out.

0. Never 1. Sometimes 2. Most of the time 3. Always

15. Corona has become a nightmare for me.

0. Never 1. Sometimes 2. Most of the time 3. Always

16. My physical activity has decreased due to the fear of Corona.

0. Never 1. Sometimes 2. Most of the time 3. Always

17. It is difficult for me to talk about Corona with others.

0. Never 1. Sometimes 2. Most of the time 3. Always

18. I get a heartbeat when I think about Corona.

0. Never 1. Sometimes 2. Most of the time 3. Always

**Obsessive questions**

1-When I touch money; I feel my hands get dirty.

1. Not at all 1. A little 2. Relatively much 3. A lot 4. Too much

2- I think even minor contact with body secretions (sweat, saliva, urine, etc.) may contaminate my clothes or harm me in any way.

1. Not at all 1. A little 2. Relatively much 3. A lot 4. Too much

3- It is difficult for me to touch something that strangers or certain people have touched.

1. Not at all 1. A little 2. Relatively much 3. A lot 4. Too much

4- It's hard for me to touch the trash or dirty things.

1. Not at all 1. A little 2. Relatively much 3. A lot 4. Too much

5- I do not use public toilets for fear of contamination and disease.

1. Not at all 1. A little 2. Relatively much 3. A lot 4. Too much

6- I do not use public telephones for fear of spreading the disease.

1. Not at all 1. A little 2. Relatively much 3. A lot 4. Too much

7- I wash my hands too long and longer than necessary.

1. Not at all 1. A little 2. Relatively much 3. A lot 4. Too much

8- Sometimes I have to wash or clean myself just because I may think I am dirty or soiled.

1. Not at all 1. A little 2. Relatively much 3. A lot 4. Too much

9- If I touch something I think is contaminated, I have to wash or clean myself immediately.

0. Not at all 1. A little 2. Relatively much 3. A lot 4. Too much
